# What has changed with subcutaneous immunotherapy against house dust mites? Eight-year, single-center real-world data

**DOI:** 10.5415/apallergy.0000000000000202

**Published:** 2025-03-31

**Authors:** Lida Bulbul, Ali Toprak, Mustafa A. Nursoy

**Affiliations:** 1 Department of Pediatric Allergy and Immunology, University of Health Science, Bagcilar Education and Research Hospital, Istanbul, Turkey; 2 Department of Biostatistics, Bezmialem Vakif University, Faculty of Medicine, Istanbul, Turkey; 3 Department of Pediatric Allergy and Immunology, Bezmialem Vakif University, Faculty of Medicine, Istanbul, Turkey

**Keywords:** Children, effectiveness, house dust mites, subcutaneous immunotherapy, systemic adverse effects

## Abstract

**Introduction:**

We aimed to investigate clinical and laboratory parameters that can predict clinical response in patients who completed subcutaneous immunotherapy (SCIT) against house dust mites (HDM) and to determine parameters associated with systemic adverse effects (SAE).

**Methods:**

In total, 108 patients who had complete medical data were evaluated.

**Results:**

Around 54.6% of patients were male; mean age was 11.7 ± 3.1 years. All patients had allergic rhinitis, 86% had asthma, and 22% had atopic dermatitis. After SCIT, mean symptom score (SS) for allergic rhinitis had decreased in 86% of patients, asthma severity had decreased in 35.5% of asthma patients. After SCIT, median eosinophil count and HDM skin prick test (SPT) diameters decreased, there was not statistically significant change in total IgE (*P* = 0.001, 0.001, and 0.824, respectively). No statistically significant difference was detected between the SS change groups (decreased, same, or increased) in terms of age, gender, disease duration, presence and severity of asthma, presence of atopic dermatitis, duration of SCIT, time after SCIT, initial sensitization status, total IgE, eosinophil and basophil counts, and SPT diameter for HDM. However, in the group with decreasing SS, total IgE change (in direction of decrease) was found to be statistically significantly higher (*P* = 0.008). SAEs were observed in 15.7% of patients, 0.41% of injections. SAEs were more common in girls (*P* = 0.023). Initial eosinophil count, basophil count, and dermatophagoides farina SPT diameter were statistically significantly higher in group with SAE (*P* = 0.007, 0.008, and 0.036, respectively).

**Conclusion:**

HDM-specific SCIT is a treatment that provides reduction in allergic rhinitis symptoms and asthma severity in children. However, we could not identify any clinical or laboratory findings that could predict clinical success before treatment. Girls and patients with high eosinophil and basophil counts should be monitored more carefully for the development of SAE.

## 1. Introduction

Allergen-specific immunotherapy (AIT) is a treatment method that has been used for more than a century for the treatment of IgE-mediated allergic diseases. It is the only treatment method that changes the direction of the immune system by gradually increasing the repeated administration of high-dose allergens to allergic patients and enabling the development of tolerance by targeting pathogenesis [1, 2]. Allergic rhinitis (AR) and/or conjunctivitis, asthma, bee venom allergies, and food allergies are the most common indications [3]. Subcutaneous, sublingual, and oral immunotherapy are the most used methods.

Subcutaneous immunotherapy (SCIT) is the oldest immunotherapy method and has been used since 1911 [4]. The effectiveness of SCIT has been demonstrated in multiple randomized controlled trials in AR patients with moderate-to-severe symptoms despite appropriate pharmacotherapy. It is also recommended by guidelines for the treatment of AR in childhood [5, 6]. There are studies showing that it prevents new sensitizations and the development of asthma [7, 8]. Global Initiative for Asthma (GINA) recommends SCIT as a treatment method for children and adults with asthma [9]. With SCIT, improvement in asthma symptom scores and medication scores, also a decrease in bronchial hyperreactivity has been reported [10, 11].

In this study, we primarily aimed to compare the clinical and laboratory findings before and after treatment in patients who completed SCIT treatment against house dust mites (HDM) for 3 to 5 years to investigate the clinical and laboratory parameters that can predict good clinical response. Second, we aimed to determine the frequency of systemic adverse effects (SAE) during treatment, the risk factors that may be associated with SAE, and the clinical and laboratory parameters that can predict systemic reactions.

## 2. Materials and methods

### 2.1. Study design, study population, and data collection

The files of 295 patients with AR and/or allergic asthma followed by Bezmialem Vakif University Pediatric Allergy and Immunology Clinic and who started SCIT between 2016 and 2022 were retrospectively evaluated. Demographic and medical information was obtained from patient files and hospital automation records. Immunotherapy was terminated in less than 3 years for various reasons in 141 patients. Treatment of 148 patients was completed by administering SCIT for 3 to 5 years. Of these 148 patients, 26 were excluded from the study because their post-SCIT examinations were incomplete. The remaining 122 patients and/or their parents were contacted by the researcher by telephone. In total, 108 patients who could be interviewed, gave their consent to the study and had complete medical data were included in the study.

AR was diagnosed in patients with clinical symptoms (2 or more of the symptoms of, rhinorrhea, sneezing, nasal itching, and nasal congestion and/or eye complaints) accompanied by a positive skin prick test (SPT) compatible with the clinical symptoms [12]. Asthma was diagnosed according to GINA recommendations [9]. Asthma severity classification was made in asthmatic patients according to the asthma control treatments they received at the beginning of SCIT and is currently, in line with GINA recommendations [9].

In all patients, demographic and clinical information (gender, age, and disease duration at the beginning of SCIT, diagnosis, and asthma severity in asthmatic patients), laboratory findings at the beginning of SCIT, and 3 to 12 months after the end of SCIT (Skin test results, serum total IgE level, blood total leukocyte count, absolute eosinophil values, and eosinophil and basophil values) were recorded from patient files. In SPT, swelling values ≥3 mm were considered positive. The average of the largest diameter and the largest vertical diameter of the swelling in the test was recorded as the result. In addition, the sum of the SPT wheal diameters against Dermatophagoides pteronyssinus (DP) and Dermatophagoides farina (DF) was recorded as house dust total SPT diameter. Patients who were sensitive only to DP and/or DF antigens were grouped as monosensitized, and patients with multiple sensitivities were grouped as polysensitized. Related to SCIT treatment; the duration of treatment, the type of allergen extract, SAEs, and the reasons for discontinuing SCIT were noted. SAEs were classified according to the World Allergy Organization’s grading system, revised in 2017 [13]. Local side effects could not be evaluated because their records were missing from the files.

### 2.2. Patient interviews

A telephone interview was conducted with patients and/or parents. During the interview, after obtaining informed consent from patients and/or families, they were asked to score their AR complaints at the beginning of SCIT and at the present time, considering all symptoms such as runny nose, nasal itching, sneezing, nasal congestion, and/or eye complaints (0: none at all, 10: very severe, significantly affecting daily life and sleep quality). Medication use for AR and asthma was recorded. Patients with atopic dermatitis (AD) were asked whether there was a decrease in skin symptoms.

### 2.3. Subcutaneous immunotherapy

For SCIT, a standardized depot ALUTARD SQ (Dermatophagoides mix) was used. Before and after the injections, patients were questioned about their symptoms and were examined by a pediatric allergist. Injections were administered by 2 experienced nurses working in the Pediatric Allergy Department. The classic protocol was used in the buildup phase for all patients. Injections were administered initially once a week during the buildup phase, with a gradually increasing dosage schedule. In the maintenance phase, injections were administered at the maximum tolerated dose with intervals of 4 to 6 weeks. Patients who continued SCIT for 3 to 5 years were considered to have completed their treatment.

### 2.4. Ethical statement

This research was conducted ethically in accordance with the World Medical Association Declaration of Helsinki. The clinical protocol was approved by the Ethical Committee of the Bezmialem Vakif University (approval No. 2023/305). Informed consent was obtained from all parents and/or patients.

### 2.5. Statistics

Statistical evaluation was performed using IBM SPSS Version 25.0 (IBM SPSS Statistics for Windows, Version 25.0. Armonk, NY: IBM Corp, Chicago, USA). In the study, median, minimum, and maximum values, numbers (n), and percentages (%) were used for descriptive data. The chi-square test was used for the analysis of the categorical data. The conformity of continuous variables to normal distribution was examined by visual (histogram and probability charts) and analytical methods (Kolmogorov–Smirnov/Shapiro–Wilk tests). The Mann–Whitney *U* test was used for the comparison of more than 2 groups, which did not fit the normal distribution. The investigation for a prognostic cutoff value is based on receiver-operating characteristic (ROC) curves. The Wilcoxon Signed Ranks Test was used for with-in-group analysis. The statistical significance level was determined as *P* < 0.05.

## 3. Results

**Figure 1.**
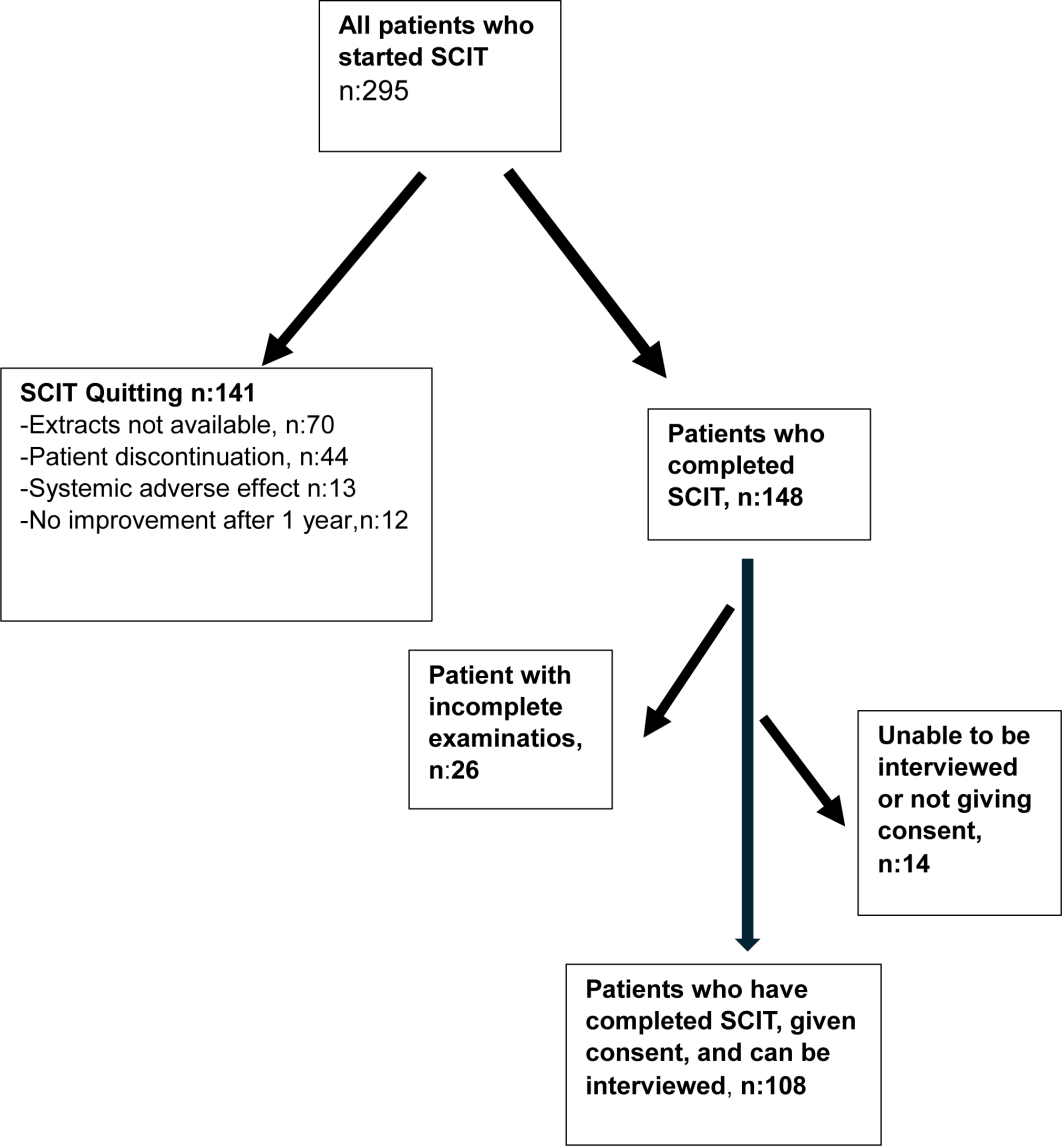
Flowchart of patient selection. SCIT, subcutaneous immunotherapy.

The study included 108 patients who completed SCIT treatment, a flowchart showing patient selection for the study was given in Figure 1. The total number of injections administered was 7702. Around 54.6% (n: 59) of our patients were male, and the mean age was 11.7 ± 3.1 years (min: 5, max: 18 years, median: 12 years, Q1–Q3: 9–14 years).

All our patients had a diagnosis of AR, most of them had a diagnosis of asthma (n: 93, 86.1%), and the most common diagnosis group was asthma-AR (n: 73, 67.1%). AD was also diagnosed in 22.2% of our patients (n: 24). Mean disease duration was 6.2 ± 2.6 years (min-max: 2–14 years, median: 6 years, Q1–Q3: 4–7 years). Of the 93 patients with a diagnosis of asthma, 49 (52.7%) had mild asthma. The mean time since the end of the SCIT was 2.7 ± 1.5 (min: 6 months, max: 6 years) years. Demographic and clinical characteristics of the patients are given in Table 1.

**Table 1.** Demographic and clinical characteristics of the patients

		n	% within subgroup	% within the whole group
Gender	Female	49		45.4
	Male	59		54.6
Diagnosis	AR+asthma	73		67.1
	AR	14		13
	AR+asthma+AD	23		18.5
	AR+AD	1		0.9
Asthma severity (pre-SCIT) subgroup n: 93	Mild	49	52.7	45.4
	Moderate	44	47.3	40.7
Asthma severity (post-SCIT) n: 93	Mild	80	86	74.1
	Moderate	13	14	12.0
History of food allergy	No	105		97.2
	Yes	3		2.8
Sensitization	Monosensitization	92		85.2
	Polysensitization	16		14.8
Initially polysensitized subgroup n:16	HDM+animal dander	7	43.8	6.5
	Mite +pollen	5	31.3	4.6
	Mite +pollen+ animal dander	3	18.8	2.8
	Mite+ cockroach	1	5.3	0.9
Animal dander sensitized subgroup n:10	Cat	10	100	9.3
	Dog	2	20	1.9
SCIT duration	3–4 years	25		23.1
	4–5 years	83		76.9
SAE	No	91		84.3
	Yes	17		15.7
SAE type (grade*) subgroup n:17	Bronchoconstriction (grade 3)	14	82.3	13
	Upper respiratory tract symptoms + bronchoconstriction (grade 3)	2	11.8	1.8
	Upper respiratory tract symptoms	1	5.9	0.9
SAE period subgroup n:17	Buildup	3	17.6	
	Maintenance	12	70.6	
	Both of	2	11.8	
Number of SAE Subgroup n:17	1 time	10	58.8	
	2 times	2	11.8	
	≥3 times	5	29.4	
SS change after SCIT	Same or increased	15		13.9
	Decreased by 1–3 points	28		25.9
	Decreased by 4–6 points	39		36.1
	Decreased by 7–10 points	26		24.1
Asthma severity change after SCIT	Same	58	62.4	53.7
	Decreased	33	35.5	30.6
	Increased	2	2.1	1.8
HDM SPT diameter change after SCIT	Decreased	77		71.3
	Same or increased	31		28.7
Post-SCIT SPT negative	DF <3 mm	1		0.9
	DP <3 mm	None		
Decrease in AD severity n:24	Yes	16		66.7
	No	8		33.3
New sensitization after SCIT	No	75		69.4
	Yes	33		30.6
New sensitization type after SCIT n:33	Pollen	23	69.7	21.3
	Animal dander	14	42.4	12.9
	Cockroach	2	6.1	1.9
Time after SCIT	<1 year	12		11.1
	≥1 year	96		88.9
Medication for AR after SCIT	None	59		54.6
	Intermittent	33		30.6
	Often or constantly	16		14.8

* Systemic adverse reactions were classified according to the World Allergy Organization’s grading system, revised in 2017 (13).

AD, atopic dermatitis; AR, allergic rhinitis; DF, dermatophagoides farina; DP: dermatophagoides pteronyssinus; HDM, house dust mites; SAE, systemic advers effect; SCIT, subcutaneous immunotherapy; SPT, skin prick test.

Most patients (n: 92, 85.2%) were initially monosensitized. All monosensitized patients were HDM-sensitive. The accompanying sensitizations in 16 polysensitized patients are shown in Table 1. SCIT was administered against HDM in all patients.

Patients’ pre-SCIT and current symptom scores for AR were evaluated. The mean symptom score was 8.8 ± 1.2 (min-max: 5–10) before SCIT and 4.5 ± 2.8 (min-max: 0–10) after SCIT. The symptom score decreased in 86.1% (n: 93) of the patients and remained the same or increased in 13.9% (n: 15). The mean score decrease in the group with decreased symptom scores was 5.2 ± 2.3 points. Sixteen patients (14.8%) and/or their parents reported that AR symptoms decreased in the first years after SCIT but increased again over time. The duration of post-SCIT in this patient group ranged from 2 to 4 years. In 13 of these patients, current symptom scores decreased compared to pre-SCIT (min: 1 point, max: 8 points, mean: 4.5 ± 2.4). In 2 patients, pre-SCIT and current symptom scores were the same (8 points in 1 patient, 9 points in the other), and in 1 patient, they were worse (pre-SCIT: 8 points, current: 10 points, at the end of SCIT: zero points). While 14 of these patients (85.2%) were initially monosensitized, 2 (14.8%) were polysensitized.

In the current asthma severity assessment of the patients after SCIT, 80 (86%) patients had mild asthma, and 13 (14%) patients had moderate asthma. Asthma severity was the same in 58 patients (62.4%) as before SCIT. Fifty of these patients had mild asthma before and after SCIT and 8 patients had moderate asthma, which continued at the same severity after SCIT. Asthma severity decreased in 33 patients (35.5%) and asthma severity increased in only 2 patients (2.2%). Only 1 of the 15 patients who did not have asthma before SCIT developed asthma symptoms after SCIT.

16 of 24 patients (66.7%) with AD had a decrease in skin symptoms compared with before SCIT.

Laboratory data of the patients at the beginning of SCIT and 3 to 12 months after the end of SCIT were compared. There was a decrease in the mean and median blood eosinophil count and percentage, SPT diameters, and symptom scores, there was no statistically significant change in the values of mean and median serum total IgE, blood basophil count, and percentage (Table 2). It was observed that the total diameter of HDM SPT decreased in most patients (n: 77, 71.3%), but SPT was negative in only 1 patient (DF: 2 mm, DP: 3 mm in the relevant patient). In the group with decreased SPT diameter, the mean total diameter decrease was 5.8 ± 4.6 mm (min-max: 1–21 mm, median: 5 mm, Q1–Q3: 2–8 mm). The results of changes in SPT diameter, total serum IgE, and blood eosinophil count are given in detail in Table 3.

**Table 2.** Laboratory values and symptom scores before and after SCIT

	Pre-SCIT		After SCIT		
	Mean ± SD (min-max)	Median (Q1–Q3)	Mean ± SD (min-max)	Median (Q1–Q3)	*P* *
Semptom score	8.8 ± 1.2 (4–10)	9 (8–10)	4.5 ± 2.8 (0–10)	4 (2–6)	<0.001
Total IgE (IU/ml)	513 ± 665 (13–3987)	294 (130–564)	569 ± 911 (13–6870)	296 (118–670)	0.824
Eosinophil count, cell/mm³	604 ± 421 (10–2190)	530 (343–738)	338 ± 235 (17–1110)	275 (170–472)	<0.001
Basophil count, cell/mm³	75 ± 42 (0–240)	74 (40–100)	75 ± 35 (10–160)	70 (40–100)	0.932
HDM total diameter, mm	17.1 ± 6 (7–34)	16 (13–21)	14.1 ± 5.1 (5–33)	13 (11–16)	<0.001
DF SPT diameter (mm)	8.5 ± 3.9 (3–20)	7 (6–10)	7.1 ± 2.8 (2–17)	6 (5–8)	0.001
DP SPT diameter (mm)	8.6 ± 3 (4–17)	8 (6–10)	7 ± 2.7 (3–16)	6 (5–8)	<0.001

DF, Dermatophagoides farina; DP, Dermatophagoides pteronyssinus; HDM, house dust mites; SCIT, subcutaneous allergen immunotherapy; SPT, Skin prick test.

* Wilcoxon Signed Ranks test.

**Table 3.** SPT diameter, total serum IgE and blood eosinophil count SS changes before and after SCIT

	n (%)	Mean ± sd (min-max)	Median (Q1–Q3)
HDM total diameter decrease/mm	77 (71.3)	5.8 ± 4.6 (1–21)	5 (2–8)
HDM total diameter increase/mm	28 (25.9)	4.3 ± 3.4 (1–16)	3 (1.25–6)
HDM total diameter same	3 (2.8)		
DF decrease/mm	64 (59.3)	3.8 ± 3.3 (1–15)	3 (1–5)
DF increase/mm	30 (27.8)	3 ± 2.2 (1–8)	2 (1–4)
DF same/mm	14 (12.9)		
DP decrease/mm	73 (67.6)	3.2 ± 2.1 (1–10)	3 (1–4)
DP increase/mm	17 (15.7)	3.4 ± 2.1 (1–8)	3 (1–5)
DP same/mm	18 (16.7)		
Total IgE change/IU/ml	108 (100)	−55 ± 570 (−2883 to 1448)	2 (−178 to 150)
Eosinophil count change	108 (100)	271 ± 352 (−810 to 1410)	230 (65–427)

DF, Dermatophagoides farina; DP, Dermatophagoides pteronyssinus; HDM, house dust mites.

There was no statistically significant difference between the after SCIT SS change groups (decreased, same, or increased) in terms of age, gender, disease duration, presence of asthma, severity of asthma, presence of AD, duration of SCIT, initial sensitization status, after SCIT time, initial serum median total IgE, median blood eosinophil and basophil count, and SPT diameter values for DF and DP, eosinophil count change. However, in the group with decreasing SS, the serum total IgE change (in the direction of decrease) was found to be statistically significantly higher (*P* = 0.008).

There was no statistically significant difference between the HDM SPT diameter change groups after SCIT (decreased, same, or increased); age, disease duration, presence of asthma, severity of asthma, presence of AD, duration of SCIT, initial sensitization status, after SCIT time, initial median serum total IgE, median blood eosinophil and basophil count, and SPT diameter values for DF and DP, eosinophil count change and serum total IgE change. The same or increased HDM SPT diameter rate was higher in boys (39%) than in girls (16.3%) (*P* = 0.011). In the decreased HDM SPT diameter group, initial DF and DF diameters were higher at the advanced significance level (*P* < 0.001 for both comparisons).

New sensitizations were detected in approximately one-third of the patients in the SPT after SCIT (n: 33, 30.6%). Among the patients with new sensitization, 25 (75.8%) were initially monosensitized, while 8 (24.2%) were polysensitized. The most frequently added sensitizations were pollen (n: 23, 69.7%) and animal dander (n: 14, 42%) antigen sensitization. There was no statistically significant difference between patients with and without new sensitization in terms of age, gender, disease duration, presence of asthma, severity of asthma, presence of AD, duration of SCIT, initial sensitization status, initial serum median total IgE, median blood eosinophil and basophil count, SPT diameter values for DF and DP, eosinophil count change. It was determined that the median change value of serum total IgE in the group that developed new sensitization was higher—in the direction of increase—than in the group that did not develop new sensitization (*P* = 0.031). Patient groups with and without new sensitization were compared in terms of SS decrease status (decreased, increased, or the same) and the amount of SS decrease (in patients with SS decrease), there was no statistically significant difference between the two groups (*P* = 0.546, *P* = 0.250, respectively).

The relationship between SS change, HDM SPT change, new sensitization status and demographics, and clinical and laboratory findings are given in Table 4.

**Table 4.** The relationship between SS change, HDM SPT change, new sensitization status and demographic, clinical and laboratory findings

	SS change		*P*	HDM diameter change		*P*	New sensitization		*P*
	Decreased (n:93)	Same or increased (n:15)		Decreased (n:77)	Same or increased (n:31)		No (n:75)	Yes (n:33)	
Gender n (%) Female (n:49) Male (n:59)	43 (87.8) 50 (84.7)	6 (12.2) 9 (15.3)	0.065*	41 (83.7) 36 (61)	8 (16.3) 23 (39)	0.011*	37 (75.5) 38 (64.4)	12 (24.5) 21 (35.6)	0.212
Age (years) mean ± SD	11.7 ± 3.2	11.8 ± 2.9	0.869†	11.9 ± 3.4	11.1 ± 2.4	0.222†	12 ± 3.2	11 ± 2.9	0.136
Disease duration (years)mean ± SD	6.3 ± 2.7	5.5 ± 2.3	0.325†	6.4 ± 2.7	5.6 ± 2.4	0.150†	6.3 ± 2.6	5.8 ± 2.7	0.421
After SCIT time (years) mean ± SD	2.7 ± 1.6	3 ± 1.4	0.41†						
Asthma n (%) With (n:93) Without (n:15)	79 (85) 14 (93.3)	14 (15) 1 (6.7)	0.689*	67 (72) 10 (66.7)	26 (28) 5 (33.3)	0.760*	66 (71) 9 (60)	27 (29) 6 (40)	0.384
Asthma severity n (%) Mild (n:49) Moderate (n:44)	43 (87.8) 36 (81.8)	6 (12.2) 8 (18.2)	0.424	32 (65.3) 35 (79.5)	17 (34.7) 9 (20.5)	0.127*	33 (67.3) 33 (75)	16 (32.7) 11 (25)	0.417
AD n (%) With (n:24) Without (n:78)	22 (91.7) 66 (84.6)	2 (8.3) 12 (15.4)	0.51	16 (66.7) 58 (74.4)	8 (33.3) 20 (25.6)	0.460*	52 (66.7) 18 (75)	26 (33.3) 6 (25)	0.442
Initially sensitization n (%) Monosensitized n:92 Polysensitized n:16	80 (87) 13 (81.2)	12 (13) 3 (18.8)	0.694*	66 (71.7) 11 (68.7)	26 (28.3) 5 (31.3)	0.773*	67 (72.8) 8 (50)	25 (27.2) 8 (50)	0.082*
SCIT duration 3–4 years n:25 4–5 years n:83	24 (96) 69 (83.1)	1 (4) 14 (16.9)	0.184	16 (64) 61 (73.5)	9 (36) 22 (26.5)	0.358	17 (68) 58 (69.9)	8 (32) 25 (30.1)	0.858
Total IgE IU/ml (mean ± SD) Median (Q1–Q3)	524 ± 603 304 (140–661)	444 ± 689 164 (103–294)	0.054‡	505 ± 597 300 (149–596)	533 ± 818 187 (126–447)	0.342	498 ± 623 294 (132–622)	549 ± 760 294 (126–512)	0.923‡
Eosinophil count (mean ± SD) Median (Q1–Q3)	622 ± 441 540 (354–765)	483 ± 224 500 (308–668)	0.355‡	606 ± 425 540 (320–800)	596 ± 414 500 (380–710)	0.852	637 ± 440 560 (340–830)	526 ± 366 500 (324–675)	0.226‡
Basophil count (mean ± SD) Median (Q1–Q3)	76 ± 43 80 (41–100)	62 ± 26 60 (40–81)	0.272‡	74 ± 40 71 (41–100)	77 ± 46 80 (40–110)	0.605	72 ± 41 68 (40–100)	82 ± 44 80 (55–100)	0.242‡
DF SPT diameter mm (mean ± SD) Median (Q1–Q3)	8.7 ± 4 8 (6–10)	7.3 ± 2.6 7 (5–10)	0.319‡	9.5 ± 4 9 (6–12)	6.2 ± 2.3 6 (5–7)	<0.001	8.6 ± 3.3 8 (6–10)	8.5 ± 4.1 7 (6–10)	0.835‡
DP SPT diameter mm (mean ± SD) Median (Q1–Q3)	8.6 ± 3 8 (6–10)	8.3 ± 3 8 (7–9.5)	0.651‡	9.3 ± 3 9 (7–10)	6.9 ± 2.1 6 (5–8)	<0.001	8.5 ± 2.9 8 (6–10)	8.7 ± 3.1 9 (6–10)	0.912‡
Total IgE change IU/ml (mean ± SD) Median (Q1–Q3)	8 ± 478 17 (−124 to 174)	−449 ± 886 −46 (−533 to −18)]	0.008‡	−63 ± 500 3(−140 to 146]	−34 ± 722 1(−300 to 176)	0.908‡	21 ± 522 9 (−87 to 173)	(−227) ± 641 −46 (−473 to 67)	0.031‡
Eosinophil count change (mean ± SD) Median (Q1–Q3)	288 ± 364 240 (90–440)	163 ± 251 190 (−120 to 340)	0.190‡	257 ± 343 240 (54–425)	306 ± 377 200 (120–540)	0.742‡	291 ± 370 240 (57–450)	224 ± 307 210 (80–385)	0.596‡

AD, atopic dermatitis; DF, dermatophagoides farinae; DP, dermatophagoides pteronyssinus; SCIT, subcutaneous allergen immunotherapy; SD, standard deviation; SPT, skin prick test.

* Chi-square tests.

† Independent samples test.

‡ Mann-Whitney test.

**Figure 2.**
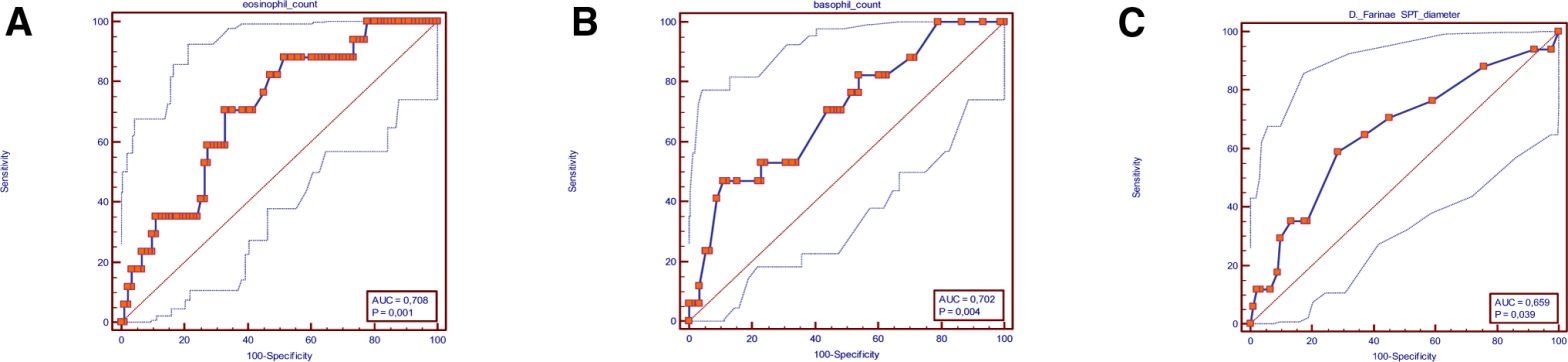
ROC analyzes, to predict systemic adverse effects using the variables eosinophil count **(A**), basophil count **(B**), and **(D**). Farinae SPT diameter **(C**). ROC, receiver-operating characteristic; SPT, skin prick test.

During SCIT, SAEs were observed in 15.7% of patients (n: 17), and 0.41% of injections (32 of 7702 total injections). SAEs did not recur in most patients with subsequent doses (n: 10, 58.8%). In our patients, according to the World Allergy Organization 2017 classification, grade 4 and 5 reactions were not observed. Detailed results regarding the observed SAEs are given in Table 1. The next SCIT allergen dose was reduced in all patients with SAEs. There was no statistically significant difference between the groups with and without SAE during treatment in terms of age and disease duration. When gender distribution was evaluated, the rate of SAE in female patients (n: 12, 24.5%) was statistically significantly higher than in male patients (n: 5, 8.5%) (*P* = 0.023). While SAEs were not observed in any of the 15 patients without asthma diagnosis, they were observed in approximately one-fifth of asthmatic patients (n: 17, 18.3%); however, the difference was not statistically significant (*P* = 0.121). There was no statistically significant difference in the development of SAEs between the mild and moderate asthmatic groups. When the patient groups with and without SAE were compared in terms of laboratory values at the beginning of treatment, there was no statistically significant difference between the 2 groups in terms of total IgE, leukocyte count, sensitization status, and SPT diameter values for HDM. Initial eosinophil and basophil, DF SPT diameter was statistically significantly higher in the group with SAE (*P* = 0.007, *P* = 0.008, *P* = 0.036, respectively). A comparison of clinical and laboratory findings of patients with and without SAE is given in Table 5. ROC analyses were performed to predict SAEs using the variables eosinophil count, basophil count, and DF SPT diameter. In the ROC analysis for the eosinophil count, a cutoff of >590 cells/mm^3^ was found with 70.6% sensitivity and 67% specificity (AUC = 0.708 ± 0.06; *P* = 0.001). In the ROC analysis for the basophil count, 47.1% sensitivity, 89 % specificity, and cutoff >119 cells/mm^3^ were found (AUC = 0.702 ± 0.07; *P* = 0.004). In the ROC analysis for the DF SPT diameter, 58.8% sensitivity, 71.4% specificity, and cutoff >9 mm were detected (AUC = 0.659 ± 0.08; *P* = 0.039) (Fig. 2).

**Table 5.** Comparison of clinical and laboratory findings of patients with and without SAE

	Systemic adverse effects		
	No (n:90)	Yes (n:17)	*P*
Gender n (%) Female (n:49) Male (n:59)	37 (75.5) 54 (91.5)	12 (24.5) 5 (8.5)	0.023*
Age (years) mean ± SD median (Q1–Q3)	11.6 ± 3.1 12 (9–14)	12 ± 3.4 12 (8.5–14.5)	0.659†
Disease duration (years) mean ± SD median (Q1–Q3)	6.2 ± 2.6 6 (4–7)	5.8 ± 2.7 5 (4–7)	0.537†
Asthma n (%) With (n:93) Without (n:15)	76 (81.7) 15 (100)	17 (18.3) 0 (0)	0.121*
Asthma severity n (%) Mild (n:49) Moderate (n:44)	39 (79.6) 37 (84.1)	10 (20.4) 7 (15.9)	0.575
Sensitization n (%) Monosensitized n:92 Polysensitized n:16	78 (84.8) 13 (81.2)	14 (15.2) 3 (18.8)	0.715*
Total IgE IU/ml Mean ± SD Median (Q1–Q3)	498 ± 679 293 (126–544)	590 ± 599 419 (178–1050)	0.358†
WBC Mean ± SD Median (Q1–Q3)	8400 ± 2226 8130 (6730–9800)	9330 ± 2832 8000 (7185–11000)	0.236†
Eosinophil count Mean ± SD Median (Q1–Q3)	556 ± 390 500 (290–720)	856 ± 497 700 (530–1115)	0.007†
Basophil count Mean ± SD Median (Q1–Q3)	69 ± 38 70 (40–90)	105 ± 51 93 (67–135)	0.008†
DF SPT diameter mm Mean ± SD Median (Q1–Q3)	8.2 ± 3.6 7 (6–10)	10.4 ± 4.5 10 (6.5–14)	0.036†
DP SPT diameter mm Mean ± SD Median (Q1–Q3)	8.5 ± 3.1 8 (6–10)	9.2 ± 2.5 9 (7–10)	0.36†

DF, Dermatophagoides farina; DP, Dermatophagoides pteronyssinus; SPT, skin prick test; WBC, white blood cell.

* Chi-square test.

† Mann–Whitney test.

## 4. Discussion

SCIT is a treatment method that has been used for many years in the treatment of AR and/or allergic asthma and can change the natural course of allergic diseases by targeting pathophysiological mechanisms [14, 15]. There is also data on the effectiveness of SCIT in AD patients with aeroallergen sensitivity [16].

SCIT is in general recommended for the treatment of AR in children and adults with moderate-to-severe disease that is suboptimally controlled despite pharmacotherapy. AIT is effective in improving symptom and medication scores during treatment in patients with allergic rhino-conjunctivitis, and there is evidence that these benefits persist after treatment discontinuation [6]. In our study, a decrease in symptom scores was observed in 86% of patients with AR. This result is consistent with the literature, including SCIT studies with HDM [17–21]. A meta-analysis evaluating the efficacy and safety of sublingual (SLIT) and SCIT in children with AR had compared 1279 patients in the SCIT group and 1223 patients in the non-SCIT group and had illustrated that compared with the non-SCIT group, the SCIT group had a significantly lower symptom scores [22]. A retrospective cohort study in Indonesia comparing clinical improvement and healthcare costs in children with AR who received SCIT and those who did not (1098 patients in each group) at 18 months follow-up reported significant improvement in symptom scores in the SCIT group [23]. In a retrospective cohort study (REACT study) examining the long-term real-world effectiveness of allergen immunotherapy (SLIT and SCIT) in patients with AR and asthma (children and adults), 46,024 subjects treated with AIT were matched to controls. A greater reduction in AR prescriptions was observed in the AIT group compared with the control group [24].

In our study, asthma severity decreased in one-third of asthmatic patients after SCIT. In half of asthmatic patients, asthma severity, which was mild before SCIT, did not increase and continued as mild. Since there was no control group in our study, we could not evaluate the effect of the decrease in severity that may occur with increasing age in asthmatic patients. HDM SCIT is recommended for adults and children with controlled HDM-driven allergic asthma as the add-on to regular asthma therapy to decrease symptoms and medication needs [25]. In a meta-analysis evaluating the effectiveness of allergen immunotherapy (SCIT and SLIT) in allergic asthma (child and adult), it was reported that significant reductions in short-term symptoms and medication scores could be achieved with AIT [26]. A Cochrane analysis published in 2003 reported that immunotherapy reduced asthma symptoms and the use of asthma medications and improved bronchial hyperreactivity [11]. In the REACT study, the AIT group had significantly greater likelihood of stepping down asthma treatment. In addition, reductions in severe asthma exacerbations, antibiotic prescriptions, hospitalizations, length of hospital stay, and pneumonia were all lower in the AIT group [24]. Similarly, a population-based cohort study had indicated that allergic asthmatics receiving AIT were less likely to experience progression of asthma severity than asthmatics not receiving AIT [27]. In the literature, HDM and SCIT studies conducted in asthmatic patients reported a decrease in asthma severity, symptom and medication scores, and the need for rescue medication [17–19, 28–35]. In a Cochrane analysis evaluating the effectiveness of SCIT in asthma, including 88 studies (42 studies for HDM SCIT, 16 pediatric studies), it was reported that immunotherapy reduced asthma symptoms and the use of asthma medications and improved bronchial hyperreactivity [11]. Jutel et al. [17], in their real-world evidence study, had evaluated the 6-year follow-up results of patients who received SCIT with HDM compared with patients who did not receive immunotherapy. It was reported that there was a greater decrease in the number of AR and asthma medication prescriptions in the SCIT group compared with the control group during the follow-up period of after at least 2 years SCIT [17]. In a study by Cantani et al. [31], which included 300 asthmatic children who received SCIT with HDM or pollen for 3 years, they reported a significantly greater reduction in asthma symptoms, medication use, and the number of asthma attacks in the SCIT group [31].

In our study, only 1 of 15 patients who did not have a diagnosis of asthma before SCIT developed asthma symptoms after SCIT. This patient was a relatively older patient (18 years old) at the time of SCIT onset. However, since there was no control group in our study and the number of patients was insufficient, we cannot conclude that SCIT can prevent the development of asthma. The benefits of SCIT, especially for children, include avoiding long-term use and side effects of medications, preventing new aeroallergen sensitization, and reducing the risk of developing asthma. Randomized controlled trials have shown that AIT (particularly with grass and birch pollen) has a beneficial effect in preventing asthma [8, 36]. In a study including 9001 birch pollen AIT allergen immunotherapy patients (SLIT and SCIT) and 45 thousand control patients, up to 6 years post-treatment, significantly reduced AR and asthma medication intake, and significantly decreased risk of new-onset asthma medication use on-treatment [37]. A meta-analysis reported that there is evidence that AIT reduces the risk of developing asthma in the short term in patients with AR, but it is unclear whether this benefit persists in the long term [26]. The real-world evidence study in Germany reported that the probability of new-onset asthma was reduced in patients who received HDM SCIT compared with the group without AIT [17].

In our study, the mean eosinophil count and HDM SPT diameters were significantly decreased compared with the pre-SCIT values. This situation is consistent with the literature [23, 34, 38–40]. There was no notable change in the mean serum total IgE level in the whole group before and after SCIT, but IgE levels after SCIT in the patient group whose symptoms decreased tended to decrease more than in the patients whose symptoms did not decrease. There are studies reporting a decrease or increase in total IgE levels after SCIT, as well as studies reporting no change [35, 39–41].

We could not detect any clinical features or laboratory findings that could predict efficacy before starting SCIT treatment. There was no statistically significant difference between the SS change groups (SS decreasing group, SS same or increasing group) in terms of initial total IgE, eosinophil count, and HDM SPT diameter values. In some studies, it has been reported that total IgE, specific IgE/total IgE ratio, and blood eosinophil count at the beginning of treatment can be used to predict a good response to HDM SCIT treatment [40, 42–44].

In our study, two-thirds of patients with AD reported a decrease in skin findings after SCIT. Objective scores for AD severity before SCIT could not obtained from the patients’ medical records, and post-SCIT assessment was made by comparing skin findings with those before SCIT according to information obtained from patients and/or parents via telephone interviews. Furthermore, since there was no control group in our study, the effect of improvement with age, which is inherent in the disease, cannot be excluded. Recently published a meta-analysis evaluating allergen immunotherapy for AD and 23 randomized controlled trials including 1957 adult and pediatric patients sensitized primarily to HDMs were evaluated in this meta-analysis; SCIT and SLIT against aeroallergens, especially HDMs, were reported to similar and importantly improve AD severity and quality of life [16], but SCIT is not recommended as a standard treatment for patients with only AD.

One of the reported positive effects of SCIT is the prevention of new sensitizations [7]. In our study, 25 of 92 patients (27.2%) who were initially monosensitized developed new sensitizations after SCIT. We could not compare this rate because we did not have a control group. Therefore, our study is methodologically inappropriate to draw conclusions about preventing new sensitizations.

In our study, SAEs were observed in 15.7% of all patients and 0.41% of the injections. Systemic reactions were successfully treated in all patients using inhaled β-2-agonists, systemic steroids, and antihistamines according to the clinical semptoms, and there was no need for epinephrine. None of the patients developed hypotension or loss of consciousness. In a recent meta-analysis, the frequency of SAE during SCIT in asthmatic children allergic to HDM was reported with a wide range from 0–10% per injection to 0–18% per patient. It was also reported that SAEs were mostly mild or moderate in severity, and there were no fatal cases [28]. These data are similar to our study. The rate of patients diagnosed with asthma in our study was high (86%), and SAEs were observed only in asthmatic patients. However, due to the small number of patients without asthma, no statistically significant difference was found between the 2 groups in terms of SAE development. In many studies, the presence of asthma and treatment with HDM extract have been stated as risk factors for the development of side effects [45–50]. High rate of asthmatic patients may explain the relatively high rate of SAEs in our study. In our study, the most frequent SAE was bronchoconstriction. This situation is in accordance with the literature [47, 48, 51–53].

The relationship between SAE and demographic and clinical findings was examined in our study, it was found that more systemic reactions were observed in girls. In the literature, there are studies reporting that adverse effects are more common in females, and there are also studies finding no relationship between systemic reactions and gender [45, 46, 52, 54, 55]. In our study, no significant difference was found in the rates of SAE between patients with mild and moderate asthma. The relationship between SAE during SCIT and asthma, especially uncontrolled asthma, is well known [46, 47, 49, 50, 56]. For SCIT, severe asthma is an absolute contraindication, and partially controlled asthma is a relative contraindication [1].

The relationship between the presence of SAE and laboratory findings was evaluated in our study; we found that patients with SAE had higher blood eosinophil and basophil counts and percentages. Eosinophils are important effector cells in IgE-mediated allergic inflammation [57]. In 2 similar studies conducted in Türkiye, it was reported that the initial eosinophil count was higher in patients who developed adverse reactions (local and systemic) during SCIT [52, 58]. There are a limited number of studies in the literature correlating blood basophil count with SAEs, which is associated with SCIT. In the study in which we evaluated all patients who underwent SCIT in our clinic (n: 295), it was found that blood eosinophil and basophil counts were higher in patients with SAE [59]. It is well known that basophils, together with mast cells, are the main effector cells of type 1 hypersensitivity reactions [50]. For this reason, it is expected to play a significant role in the formation of SAE due to SCIT. In a study, a high basophil allergen CD63 sensitivity phenotype was reported as a major indicator of severe adverse systemic reactions during the buildup phase of honeybee SCIT [60]. In this study, patient laboratory data were collected retrospectively. Therefore, IgG_4_-type blocking antibodies, and markers indicating eosinophil cell activation (such as eosinophil-derived neurotoxin, eosinophil cationic protein) which are not routinely measured before and after treatment in clinical practice, were not available in our study and their relationship with SAEs and eosinophil counts could not be evaluated. Randomized controlled studies including more patients are needed to determine whether basal basophil and eosinophil counts are risk factors for the development of SAE during SCIT and to determine critical threshold values.

## 5. Study’s limitations

Our study’s limitations include not being prospective, not having a control group, not using a standardized symptom score to clinically evaluate SCIT efficacy (for AR and AD) and not being able to measure valuable markers such as antigen-specific IgE and IgG4, regulatory cytokines, and cells that can be used to evaluate tolerance after SCIT.

## 6. Conclusion

HDM-specific SCIT is a treatment that provides a reduction in AR symptoms and asthma severity in children with AR and/or asthma. However, we could not detect any clinical or laboratory findings that could predict the clinical success of the treatment. Although SAEs may be observed during HDM SCIT, severe reactions are rare. Girls and patients with high blood eosinophil and basophil levels should be monitored more carefully for the development of SAE. More randomized controlled studies are needed to predict the good response to SCIT and the development of SAE.

## Conflicts of interest

The authors have no financial conflicts of interest.

## Author contributions

Lida Bulbul: conceptualization, writing, data collection, literature search, critical revision of the manuscript and final approval Ali Toprak: analysis and interpretation, writing. Mustafa Atilla Nursoy: conceptualization, critical review, patients manegement, data collection.
